# Polyamine metabolism in cancer: drivers of immune evasion, ferroptosis and therapy resistance

**DOI:** 10.1017/erm.2025.10026

**Published:** 2025-11-14

**Authors:** Sainavya Sree Chenna, Siva Nageswara Rao Gajula, Lakshmi Vineela Nalla

**Affiliations:** 1 Department of Pharmacology, GITAM School of Pharmacy, GITAM (Deemed to be University), Visakhapatnam, India; 2 Department of Analysis, GITAM School of Pharmacy, GITAM (Deemed to be University), Visakhapatnam, India

**Keywords:** efferocytosis, ferroptosis, hypoxia and drug resistance, immune evasion, polyamines, tumour microenvironment

## Abstract

Polyamines putrescine, spermidine and spermine are small, positively charged metabolites indispensable for DNA stabilization, chromatin remodelling, RNA translation and redox balance, with dynamic distribution across the nucleus, mitochondria and endoplasmic reticulum. In cancer, polyamine homeostasis becomes profoundly dysregulated through altered biosynthesis, degradation and transport, driving malignant phenotypes and therapy resistance. Therefore, there is an urgent need to develop precision techniques that combine polyamine metabolism with immunotherapeutic and redox-based therapies, identify biomarkers to predict therapy response and create logical combination regimens to overcome resistance. The existing literature lacks in providing a holistic view of how polyamine dynamics intersect with diverse cancer hallmarks. Thus, this review consolidates emerging evidence on the multifaceted roles of polyamines in cancer hallmarks, with a particular focus on their impact on efferocytosis, ferroptosis and the dynamics of polyploid giant cancer cells (PGCCs). Furthermore, a comprehensive evaluation of contemporary treatment approaches that focus on polyamine metabolism, including transport blockers, biosynthesis inhibitors and various polyamine analogues, was discussed. While addressing context-dependent effects of polyamines that impede therapeutic progress, our discussion also incorporates important findings from pre-clinical and clinical investigations. Going forward, this review aims to enlighten and direct future translational research by situating polyamine biology within the broader context of cancer evolution and treatment adaptation.

## Introduction

Polyamines such as spermidine, spermine and putrescine are ubiquitous, positively charged molecules that regulate essential cellular functions in all living systems. These organic cations regulate cell growth, differentiation and protein function, making them crucial to both normal physiology and disease pathogenesis. Despite being essential for maintaining cellular homeostasis, polyamines are consistently dysregulated in cancer, which fuels resistance, stress tolerance and malignancy. This dysregulation of polyamine anabolism and catabolism establishes polyamines as active causative agents of cancer rather than passive bystanders (Ref. 1). Therefore, there is an urgent need to identify new molecular targets, given the ongoing increase in the global burden (Ref. 2).

Targeting metabolic vulnerabilities, such as the polyamine addiction of many malignancies, is gaining attention because of the drawbacks of traditional anticancer medicines, which include problems with toxicity, resistance and lack of selectivity. It is essential to comprehend these metabolic abnormalities in order to create cancer treatments that work. The cellular polyamine pool may be depleted by the irreversible inhibitor of ornithine decarboxylase (ODC) and difluoromethylornthine (DFMO). However, monotherapy often triggers compensatory mechanisms for adaptation, such as increased uptake from the extracellular environment, which limits the treatment’s effectiveness. Additionally, because polyamines affect oncogenic gene expression, mitochondrial respiration, apoptotic resistance and protein stress responses, their many subcellular functions in the nucleus, mitochondria and endoplasmic reticulum make targeted suppression more difficult (Ref. 3). Furthermore, polyamines support the neoplastic programme in the nucleus by increasing chromatin compaction and modifying gene expression. They enable resistance to programmed cell death by controlling respiration and blocking apoptotic pathways within mitochondria (Ref. 4). In the endoplasmic reticulum, polyamines affect protein folding and stress responses, allowing cancer cells to manage proteotoxic burdens from rapid proliferation (Refs. 5, 6). These multifaceted roles highlight the intricate involvement of polyamines in maintaining the adaptability and survival of cancer cells.

The investigation of polyamine metabolism as a potential therapeutic target is significantly supported by the growing identification of this process as a characteristic of cancer. To enhance the depletion of internal polyamine pools, dual inhibition techniques have been devised to prevent extracellular polyamine import, such as by combining DFMO with polyamine transport inhibitors such as AMXT1501 (Ref. 7). This dual inhibition strategy is under active clinical investigation and has the potential to overcome tumour resilience to single-agent therapies. In addition to direct metabolic targeting, the interplay between polyamines and the immune microenvironment has spurred interest in combining polyamine inhibitors with immunotherapies to restore effective anti-tumour immunity. By mitigating the polyamine-driven immunosuppressive niche, such combinations may sensitize tumours to immune checkpoint blockade and other immune-based treatments (Refs. 8, 9). Additionally, new insights into the immunomodulatory functions of polyamines offer justification for combining immunotherapies with polyamine-targeted treatments, which may improve responses by reversing the immunosuppressive tumour microenvironment and improving clinical outcomes. Thus, the polyamine pathway is a thriving field of oncology research that is poised to influence future clinical cancer management, as evident in the evolving therapeutic landscape.

This review provides a comprehensive overview of the multifaceted roles of polyamines at the intersection of cellular metabolism and cancer biology. It elucidates how polyamine metabolism and transport are reprogrammed in malignancy, influencing cancer hallmarks. Through the incorporation of current research, it also emphasizes the roles that polyamines play in emerging aspects of cancer, such as ferroptosis, efferocytosis and polyploid giant cancer cells (PGCCs), as well as their effects on the tumour microenvironment and modulation of immunological and redox balance. It also highlights recent clinical advancements and upcoming prospects for precision oncology while critically analysing new therapeutic approaches that target polyamine metabolism.

## Polyamines in normal physiology and cellular homeostasis

Life at the cellular level is orchestrated by countless molecular players, yet few demonstrate the versatility and sophistication that one of them, polyamines, does. These three simple molecules serve as master regulator of cellular vitality, coordinating processes as diverse as protein synthesis, gene expression and immune modulation, while simultaneously shaping the hostile landscape of the tumour microenvironment.

Putrescine, the simplest diamine, serves as the precursor for higher-order polyamines. Spermidine is synthesized by adding an aminopropyl group to putrescine, resulting in a triamine (Ref. 10). Later, spermine, the most complex, is produced by adding another aminopropyl group to spermidine, thereby creating a tetramine (Ref. 11). Despite their modest size, at physiological pH, they enable electrostatic interactions with negatively charged biological components, such as the phosphate backbones of DNA and RNA (Ref. 12). This charge-based affinity is the key that unlocks their wide-ranging biological influence.

Within normal cells, polyamines silently direct the symphony of life. At the molecular level, polyamines regulate chromatin structure and maintain DNA integrity (Refs. 4–6) and fine-tune gene expression through the effects on transcription and translational machinery. Their control on the ion channel activity, including Na^+^/K^+^-ATPase and calcium transporters, maintains membrane potential and cellular ion gradients (Ref. 13). Polyamines also reinforce enzyme activity, support membrane integrity and govern DNA methylation, a crucial epigenetic layer controlling gene expression (Ref. 12). Yet their functions extend beyond routine maintenance, polyamines perform critical roles in cellular stress adaptation and quality control. They maintain redox homeostasis by working as antioxidants and neutralizing reactive oxygen species (ROS) generation, and preserve redox equilibrium (Ref. 14). Notably, spermidine acts as a master regulator of autophagy, promoting the removal of damaged cellular components and extending lifespan across multiple organisms (Ref. 15). Another pivotal role lies in enabling hypusination of eukaryotic initiation factor 5A (eIF5A), which influences mitochondrial respiration, energy production, cellular metabolism and cell survival (Ref. 16).

In specialized circumstances, polyamines demonstrate context-dependent activities. They perform contradictory roles in programmed cell death, exerting either pro-apoptotic or anti-apoptotic actions depending on the cellular environment and concentration. Their necessity is most evident in rapidly proliferating cells and regenerative tissues, where they fuel proliferation, differentiation and the repair of damage tissue. For instance, they sustain tissue-specific functions and defend intestinal epithelial barrier integrity, maintaining organ function and host defence (Refs. 17, 18). The gradual addition of amine groups from putrescine to spermidine to spermine enables intricate molecular interactions, expanding their functional repertoire (Refs. 14, 19). As the cell ages, this finely tuned balance begins to wane. Polyamine levels decline with age, and this decline correlates with the development of age-related pathologies. Notably, supplementation with spermidine in model organisms has been shown to restore longevity and alleviate age-related diseases, pointing to a conserved mechanism linking polyamine homeostasis to healthy ageing (Ref. 20). However, it is possible to undermine the same mechanism that maintains normal physiology. Though polyamines maintain homeostasis, cancerous cells utilize them to promote unchecked growth, enhance their ability to withstand stress, evade programmed cell death and develop resistance to treatment.

### Metabolism of polyamines

As polyamines orchestrate essential life-maintaining functions, their high adaptability calls for rigorous regulation. With too little, cells falter in growth and survival; with too much, they are prone to becoming diseased. Therefore, polyamine homeostasis is regulated through coordinated control of anabolism, degradation, catabolism and transmembrane transport in both unicellular and multicellular organisms (Ref. 21). Under normal physiological conditions, feedback systems involving salvage routes, *de novo* synthesis, and efflux closely intracellular polyamine concentrations are tightly controlled through feedback mechanisms regulate intracellular polyamine concentrations (Ref. 22). This metabolic balance ensures that polyamines remain available to support growth and homeostasis, without tipping into cytotoxic excess.

The rate-limiting step in *de novo* polyamine biosynthesis is catalysed by ODC, which decarboxylates ornithine to yield putrescine (Ref. 23). ODC activity is highly inducible, transient and strictly controlled by antizymes and antizyme inhibitors (Ref. 2). Parallel to this, s-adenosylmethionine decarboxylase (AMD1) decarboxylates s-adenosylmethionine (SAM) to yield decarboxylated SAM (dcSAM) (Ref. 24), providing the essential aminopropyl donor groups required for higher-order polyamine synthesis. Spermidine synthase (SRM) transfers an aminopropyl group from dcSAM to putrescine, generating spermidine and 5′-methylthioadenosine (MTA) (Ref. 25). Later on, spermine synthase (SMS) appends an additional aminopropyl group from dcSAM to spermidine, producing spermine and MTA. Beyond structural synthesis, spermidine supports one of the most fascinating post-translational modifications in eukaryotes, i.e., hypusination of eIF5A occurring at a conserved lysine residue mediated by deoxyhypusine synthase (DHS) and deoxyhypusine hydroxylase (DOHH), allowing for effective translation elongation, mitochondrial respiration and cellular bioenergetics (Refs. 26, 27). Disruption of this pathway impairs normal eukaryotic cell growth and can hinder tumour progression (Refs. 28–33), highlighting its central role at the crossroads of metabolism and proliferation.

**Figure 1.**
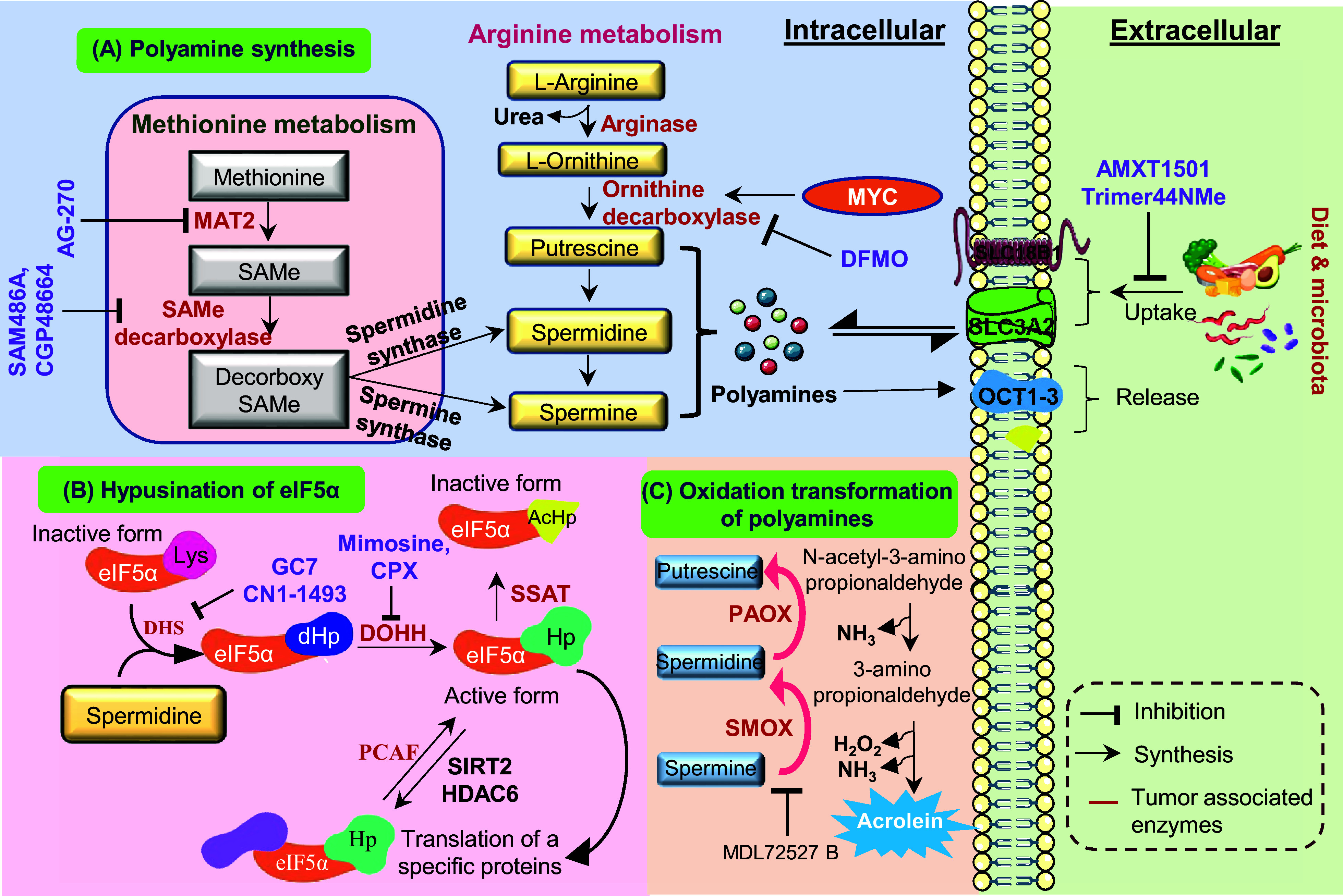
Schematic representation of polyamine metabolism, transport and eIF5A hypusination: (A) Polyamine synthesis: The methionine and arginine metabolic pathways converge to generate intracellular polyamines. Methionine is converted into S-adenosylmethionine (SAMe) by MAT, which acts as a donor for decarboxylated SAMe to fuel spermidine synthase and spermine synthase reactions. Simultaneously, arginine is metabolized by arginase to form ornithine, which undergoes decarboxylation via ornithine decarboxylase (ODC1) a proto oncogene c-Myc (MYC) regulated enzyme to produce putrescine, leading to sequential formation of spermidine and spermine. Polyamine synthesis can be inhibited by DFMO, while extracellular uptake is mediated by SLC3A2 and organic cation transporter 1 to 3 (OCT1–3), with uptake inhibitors such as AMXT1501, Trimer44NMe, and export via vesicular release. (B) Hypusination of eIF5A: Spermidine donates its aminobutyl group for post-translational modification of eIF5A, forming hypusinated eIF5A (eIF5A Hp) through sequential actions of DHS and DOHH. This active form regulates translation of proliferation-related proteins. Acetylation of eIF5A by spermidine/spermine synthase (SSAT) and P300/CBP associated factor (PCAF) or deacetylation by sirtulin-2 (SIRT2) and histone deacetylase-6 (HDAC6) dynamically controls its function, while inhibitors such as GC7, ciclopirox (CPX) and Mimosine block hypusination. (C) Oxidation and transformation of polyamines: Putrescine and spermidine undergo oxidative catabolism via PAOX and SMOX, generating acrolein, H₂O₂ and 3-amino-propionaldehyde, which contribute to oxidative stress and tumour progression. Inhibitor MDL72527B prevents this cytotoxic transformation.

To maintain equilibrium, polyamine catabolism acts as an essential counterbalance to biosynthesis. The enzyme spermidine/spermine N1-acetyltransferase (SSAT) acetylates spermidine and spermine at the N1 position, producing N1-acetylspermidine and N1-acetylspermine (Ref. 34). In order to oxidize these acetylated forms and produce hydrogen peroxide(H_2_O_2_), acetamidopropanal, putrescine (from acetylspermidine) or spermidine (from acetylspermine), SSAT is the rate-limiting enzyme that directs polyamines towards oxidation by polyamine oxidase (PAOX) (Ref. 35). An alternative route involves spermine oxidase (SMOX), which directly oxidizes spermine to spermidine, producing 3-aminopropanal and H_2_O_2_ (Ref. 36). Despite the fact that this oxidative catabolism aids in controlling intracellular concentrations, the paradoxical by-product hydrogen peroxide adds to cellular oxidative stress, especially when metabolic dysregulation or inflammation are present. The dynamic balance between biosynthetic and catabolic enzymes, coupled with regulated transport and feedback mechanisms, maintains polyamine concentrations within the narrow physiological range essential for normal cellular function (see Figure 1).

### Regulation of polyamine levels

A multi-layered network of feedback regulators, antizymes, antizyme inhibitors and coordinated transport systems is needed to maintain ideal intracellular polyamine concentrations. The antizyme family (AZ1–AZ4), which acts as a primary negative regulator of polyamine homeostasis via an autoregulatory feedback mechanism, is essential to this regulation (Ref. 37). When intracellular polyamine concentration increases, polyamines trigger a + 1 ribosomal frameshift in antizyme mRNA, producing complete functional antizyme proteins that inhibit ODC activity. These, in turn, bind ODC monomers with high affinity, preventing homodimer formation and directing the ODC-antizyme complex for ubiquitin-independent degradation by the 26S proteasome, thereby curbing putrescine synthesis (Ref. 2). They also suppress polyamine uptake, providing a secondary control point that maintains the intracellular steady state. In contrast, antizyme inhibitors (AZIN1 & AZIN2) are structurally similar to ODC but lack catalytic activity. These proteins bind antizymes and relieve their inhibition, restoring ODC activity and polyamine transport, elevating intracellular polyamine levels. High polyamine concentrations, however, downregulate AZIN1 expression, forming a negative feedback loop that reinforces polyamine-dependent suppression of ODC (Ref. 38).

The regulation of polyamines extends beyond metabolic synthesis and degradation; it also depends critically on membrane transport systems that dictate intracellular flux. Polyamine transport involves energy-dependent uptake systems that are plasma membrane potential-dependent but often sodium-independent (Ref. 39). Certain transporter proteins actively transport polyamines against concentration gradients, utilizing cellular ATP as an energy source. In specialized cell types, uptake can also occur through caveolae-mediated endocytosis, underscoring the adaptive complexity of this system. While polyamine transporters are well characterized in prokaryotes (e.g. PotABCD in *E. coli*) and lower eukaryotes (e.g. Durine permease-3 (DUR3) in *Saccharomyces*), mammalian systems remain less clearly defined. Recent discoveries, however, are beginning to fill these gaps. Notably, adenosine triphosphatase 13 isoform A4 (ATP13A4) has been identified as a putative polyamine transporter in breast cancer cells (Ref. 40), linking altered transport to oncogenic signalling. Additionally, divalent cations such as Ca^2+^ and Mg^2+^ modulate polyamine uptake dynamics, suggesting a finely tuned electrochemical coordination in mammalian cells (Ref. 41).

Polyamine export mechanisms serve as the complementary regulators, ensuring that intracellular balance does not exceed homeostatic limits. In colon cancer cells, for example, the amino acid transporter Solute carrier family-3 member 2 (SLC3A2) functions as a polyamine exporter by facilitating acetylated polyamines for extracellular arginine (Ref. 42), highlighting the coupling between polyamine turnover and amino acid metabolism. In addition, organic cation transporters (OCT1–3) also transport spermidine and spermine (Ref. 43). Model systems from *Saccharomyces cerevisiae* have been particularly instructive, identifying five polyamine export proteins, as well as Thiamine pyrophosphate transporters (TPO1–5), which provide models for understanding mammalian efflux. Within mammalian organelles, ATP13A2 (PARK9), a lysosomal transporter, exports polyamines from lysosomes to the cytoplasm, illustrating the organelle-specific regulation of polyamine distribution (Ref. 44). Importantly, the same antizymes that inhibit ODC activity also reduce polyamine uptake, thereby coupling transport control to intracellular polyamine status through a self-regulating feedback loop, Figure 1 (Ref. 37). In summary, polyamines behave distinctly across the spectrum from normal physiology to cancer progression.

Polyamines are in a state of dynamic homeostasis in normal physiology, carefully balanced to maintain cellular communication, genomic stability and controlled cell proliferation (Ref. 45). While allowing cells to flourish, this balance prevents overgrowth (Ref. 46). However, in the context of cancer, this balance is disrupted, marking one of the earliest metabolic alterations in tumourigenesis (Ref. 47). Early-stage cancers commonly upregulate polyamine biosynthesis and transport, driven by oncogenes such as MYC and RAS, to meet the heightened demands of rapid cell growth and metabolic reprogramming (Ref. 48). This metabolic reprogramming enhances intracellular polyamine pools, thereby supporting DNA synthesis, redox regulation and the translation of growth-promoting proteins (Ref. 49). As tumours evolve to advanced stages, the polyamine network acquires new layers of complexity, including epithelial-mesenchymal transition, immune evasion and therapy resistance. These stage-dependent dysregulations are often reinforced by feedback mechanisms, such as RNA editing of antizyme inhibitors, gene amplification of biosynthetic enzymes (Ref. 50), and an increased reliance on polyamine-driven post-translational modifications, like eIF5A hypusination (Ref. 33). Recognizing the dynamic, context-specific and stage-dependent roles of polyamines is crucial for designing effective targeted therapies. By addressing the vulnerabilities created by polyamine imbalance, future strategies can selectively undermine cancer cell survival without compromising normal cellular physiology. The next sections of this review explore this dramatic shift in how polyamine metabolism, once a hallmark of cellular health, becomes a cornerstone of tumourigenic adaptation.

## Integrative perspectives on polyamine-mediated regulation of cancer hallmarks

Cancer is a condition where homeostatic cellular pathways, such as polyamine metabolism, are essentially reprogrammed to support malignant expansion. One of the characteristics of many tumours is the prevalent perturbation of polyamine homeostasis, as chronically elevated intracellular concentrations in a wide variety of tumour. These metabolic deviations arise from activated biosynthetic enzymes such as ODC, AMD1, SRM and SMS, which respond to the heightened metabolic needs of proliferating cancer cells (Ref. 2). Alterations in catabolic enzymes like SMOX and PAOX are more variable and context-dependent, reflecting their dual role in tumour biology (Ref. 35). Meanwhile, disrupted expression of antizymes and antizyme inhibitors further destabilizes the regulatory network, tipping the balance towards intracellular accumulation and sustained tumour growth (Ref. 51). Beyond intrinsic synthesis, cancer cells increase polyamine uptake from the surrounding microenvironment, raising intracellular concentrations to fuel malignancy (Ref. 40). Together, these metabolic alterations in polyamine metabolism underpin their versatile roles in promoting cancer hallmarks, including cell death resistance, reprogramming tumour microenvironment and therapy resistance, which are detailed in the following section.

### Polyamines in tumour-immune interactions

The TME is a dynamic environment with many cell types. They are metabolically activated in an intricate crosstalk that determines tumour fate. Of these metabolic controllers, polyamines play a central role in immunomodulation, influencing the behaviour of immune cells, antigen presentation and cytokine signalling. Aberrant polyamine metabolism not only improves tumour cell fitness but also reprogrammes immune interactions towards immune evasion and metastasis.

#### Immune modulation

**Figure 2.**
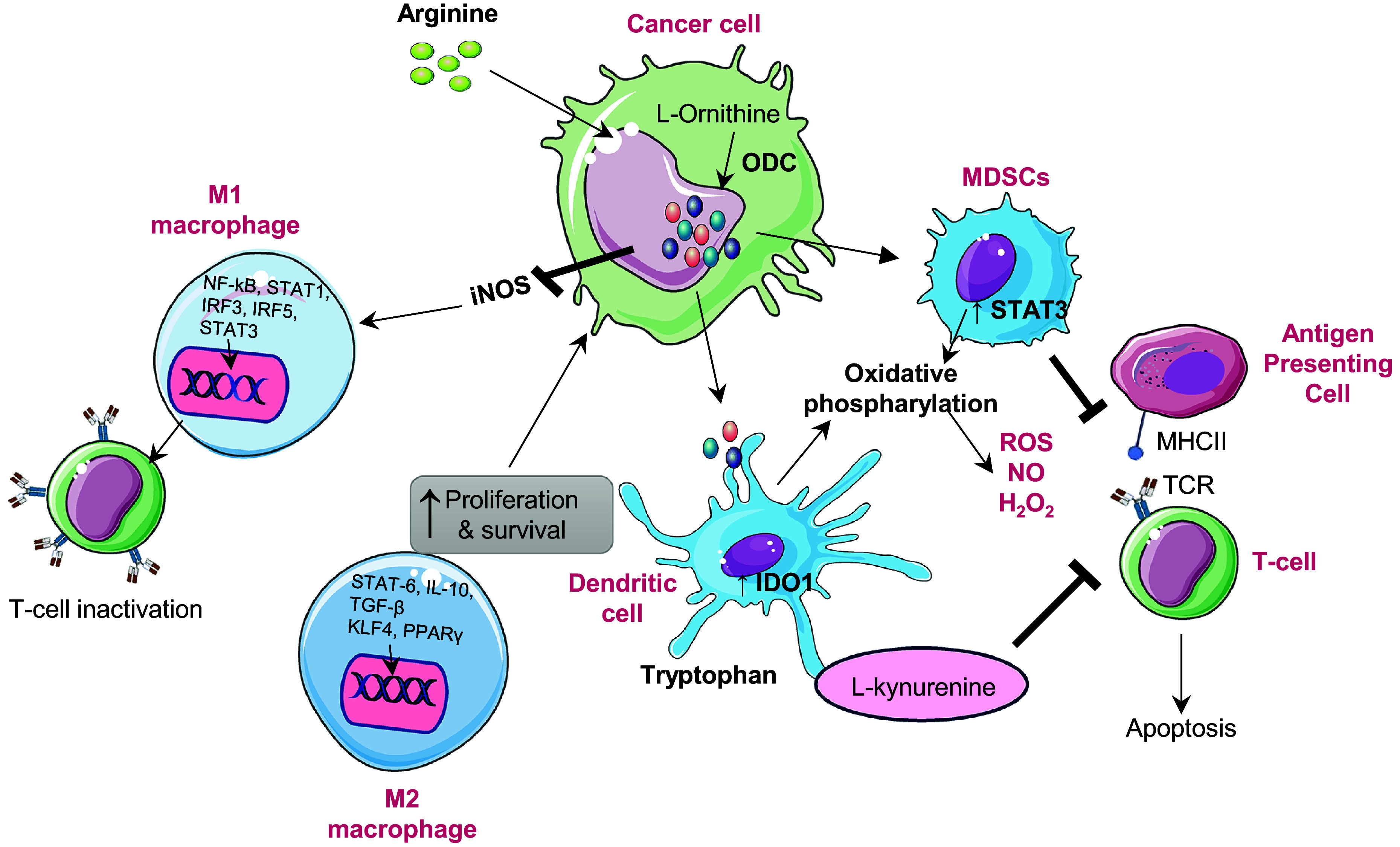
Role of polyamines in the immune modulation: Polyamine mediated immune modulation within the tumour microenvironment. Cancer cells upregulate ornithine decarboxylase (ODC1) to convert L-ornithine, derived from arginine, into polyamines, enhancing their proliferation and survival. Elevated polyamine levels enhance iNOS expression, resulting in excessive nitric oxide (NO) production. Increased NO inhibits nuclear factor kappa light chain enhancer of activated B cells (NF-kB) and STAT1 activation, leading to reduced expression of pro-inflammatory cytokines such as interleukin-12 (IL-12) and tumour necrosis factor alpha (TNF-α). This inhibition suppresses T-cell activation and drives the phenotypic shift from M1(pro inflammatory) to M2(immunosuppressive) macrophages. The M2 macrophage polarization through activation of signal transducer and activator of transcription 3 (STAT3), signal transducer and activator of transcription 6 (STAT6), peroxisome proliferator activated receptor gamma (PPARγ) and kruppel like factor-4 (KLF4), leading to increased interleukin-10 (IL-10), arginase-1 (ARG1) and inducible nitric oxide synthase (iNOS) expression. Meanwhile, myeloid derived suppressor cells (MDSCs) are activated via STAT3 signalling enhance oxidative phosphorylation, producing reactive oxygen species (ROS), nitric oxide (NO) and H_2_O_2_, which inhibit antigen presenting cell and T-cell receptor (TCR) signalling and induce T-cell apoptosis. In parallel, tumour-secreted factors enhance indolamine 2,3-dioxygenase 1(IDO1) activity in dendritic cells, converting tryptophan to L-kynurenine, which suppresses CD8^+^ T-cell activation and promotes immune tolerance. Collectively, polyamine metabolism coordinates macrophage polarization, dendritic cell reprogramming and T-cell suppression to establish an immunosuppressive microenvironment that supports tumour growth and immune evasion.

Polyamines, such as spermidine and spermine, modulate the behaviour of immune cells in the TME, influencing both innate and adaptive immunity. In many cancers, notably in glioblastoma, upregulated spermidine suppresses CD8^+^ T cell proliferation and cytokine production, impairing cytotoxic activity and facilitating tumour immune escape (Ref. 52). Moreover, polyamines reprogramme macrophage polarization, inducing the metabolic shift towards the M2 phenotype, a state characterized by anti-inflammatory and pro-tumour functions. This transition is mediated by AMPK activation and HIF-1α upregulation, both of which are driven by mitochondrial superoxide, which enhances autophagy and diminishes pro-inflammatory responses (Refs. 3, 53). In dendritic cells, polyamines also regulate immune tolerance through modulating indoleamine 2,3-dioxygenase 1 (IDO1), which contributes to T cell suppression in TME (Ref. 54). Additionally, myeloid-derived suppressor cells (MDSCs) exploit polyamines to bolster their immunosuppressive effects, Figure 2. Polyamines derived from MDSCs induce Th17 differentiation via the miR-542-5p/TGF-β/SMAD3 pathway, contributing to immune dysregulation in cancer and autoimmune diseases such as systemic lupus erythematosus (SLE) (Ref. 55).

Beyond this, activated innate lymphoid cells (ILC3) are positively regulated by putrescine, which increases IL-22 secretion, impacting mucosal immunity and inflammatory balance (Ref. 56, 57). Together, these actions illustrate that polyamines largely act as immunosuppressive mediators, dampening immune responses while maintaining tumour-promoting regulatory networks (Ref. 3, 58). However, the role of polyamines in immunity is not universally suppressive. Research evidence suggests that they also enhance immune responses under certain conditions, particularly when their levels are carefully controlled. Excessive polyamine depletion may impair the functions of immune cells, underscoring a context-dependent duality that demands precise metabolic control (Ref. 59).

Targeting polyamine metabolism holds a promising immunoregulatory strategy. Pharmacological blockade of polyamine synthesis using DFMO shown to reduce immunosuppressive cell populations (MDSCs and M2 macrophages) and enhance T-cell effector activity (Refs. 57, 60). Dual inhibition of polyamine metabolism by combining DFMO, which blocks biosynthesis, with AMXT 1501, which inhibits transport, was investigated in the phase I trial by Piha-Paul *et al.* in patients with advanced solid tumours (Ref. 61). The combination was well tolerated and resulted in disease stabilization in almost half of the patients, indicative of metabolic and immune modulatory activity. This approach mechanistically curtails intracellular polyamine pools, reduces tumour associated immunosuppression, restores CD8^+^ T-cell infiltration and promotes the transition of the tumour microenvironment from an immune ‘cold’ to an immune ‘hot’ status.

In our opinion, this clinical study reflects an emerging immunometabolic function of polyamine blockade in overcoming immune resistance. However, the modest objective response rate indicates that in some tumour types, polyamine depletion itself might not completely reverse immune suppression. This might underscore the contextual effect of polyamines, whereby while depletion may enhance anti-tumour immunity, excessive suppression could also disrupt beneficial immune functions. Thus, integration with checkpoint inhibitors or biomarker-driven approaches would be necessary for the optimization of immune modulation and therapeutic efficacy of combination with DFMO and AMXT 1501 (Ref. 7).

#### Tumour microenvironment

**Figure 3.**
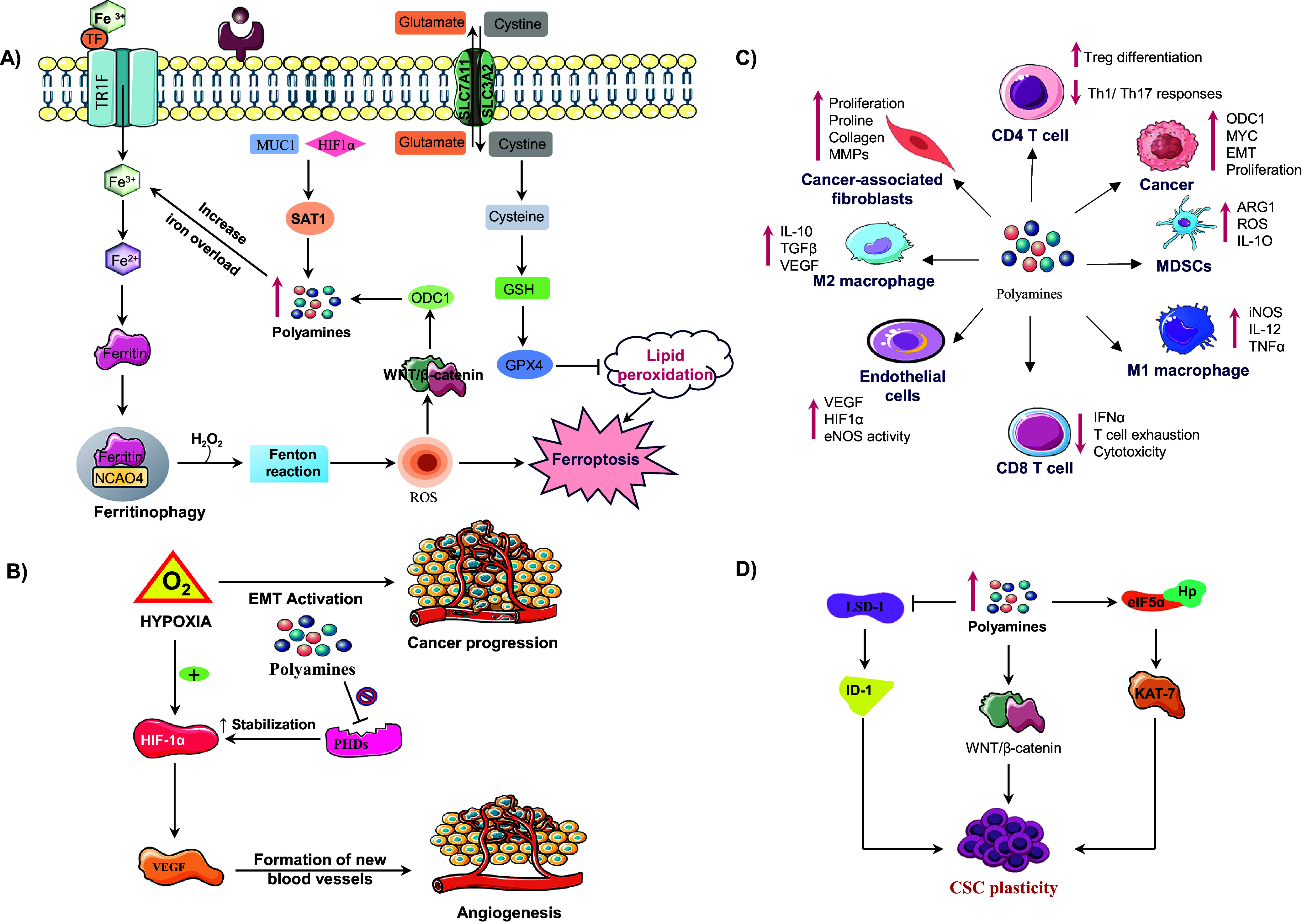
Illustration of how polyamine metabolism orchestrates cancer progression by reprogramming multiple components of the tumour microenvironment (TME). (A) Polyamine catabolism, regulated by spermidine/spermine N^1^ acetyltransferase-1 (SAT1), is influenced by factors such as mucin-1 (MUC1) and hypoxia inducible factor-1 alpha (HIF-1α). This leads to the production of toxic by-products (ROS, H₂O₂, aldehydes) via enzymes like (spermine oxidase) SMOX and peroxisomal N^1^ acetyl polyamine oxidase (PAOX), enhancing lipid peroxidation and triggering ferroptosis. Iron overload and disrupted regulation of wingless/integrated signalling pathway (Wnt/β-catenin) further exacerbate ferroptotic cell death. Upregulates ornithine decarboxylase 1 enhancing polyamine synthesis. ODC-1 activity, a key polyamine biosynthesis enzyme, is establishing a positive feedback loop. Concurrently, moderate polyamine catabolism via spermidine and SAT1/ SMOX activates glutathione peroxidase-4 (GPX4) axis, elevating glutathione (GSH) and suppressing ferroptosis by inhibiting lipid peroxidation. However, excessive polyamine peroxidation, overwhelming GPX4 defences and triggering ferroptotic cell death. Thus, polyamine metabolism acts as a redox sensitive switch between adaptive antioxidant survival and ferroptotic vulnerability in cancer cells. (B) In the tumour microenvironment elevated polyamine synthesis in cancer cells promotes an immunosuppressive and pro tumorigenic microenvironment. Polyamines induces M2 macrophage polarization, activate myeloid derived suppressor cells (MDSCs), and suppress cluster of differentiation 8 positive (CD8^+^) T-cell cytotoxicity by reducing interferon-gamma (IFN-γ) production. They also impair cluster of differentiation 4 positive (CD4^+^) T-cell activation and NK-cell function, facilitating immune evasion. In stromal cells, polyamines enhance endothelial VEGF/HIF-1α signalling, angiogenesis and CAF-mediated extracellular matrix remodelling, collectively sustaining tumour growth and metastatic progression. (C) Under hypoxic conditions, hypoxia inducible factor-1 alpha (HIF-1α) is stabilized, which promotes vascular endothelial growth factor (VEGF) mediated angiogenesis and the epithelial-mesenchymal transition (EMT), contributing to cancer metastasis. Polyamines inhibit prolyl hydroxylases (PHDs), which prevents HIF-1α degradation, thereby sustaining hypoxic signalling. (D) In CSCs, polyamines sustain cancer stem cell (CSC) self-renewal and tumour initiating potential by modulating transcriptional and epigenetic regulators. Increased intracellular polyamines inhibit lysine specific demethylase-1 (LSD-1), resulting in the upregulation of inhibitor differentiation-1(ID1), which contributes to stem cell renewal. On the other side elevated polyamine levels enhance the WNT/β catenin signalling pathway, promoting stemness associate transcriptional activation through Lin28. Polyamines also facilitate eIF5A hypusination (Hp) and activate KAT7 (lysine acetyltransferase 7) supporting the maintenance of CSC characteristics. These molecular events promote CSC plasticity.

Beyond their intracellular effects, polyamines dynamically shape the tumour microenvironment by influencing nutrient availability, signalling gradients, and intercellular communication, Figure 3C. Within this context, arginine metabolism plays a central role in regulating polyamine levels, thereby maintaining TME homeostasis and immunomodulation (Refs. 62, 63). Arginine functions in two major roles: as a key regulator of metabolic functions of immune cells and as a polyamine biosynthesis substrate. In T-cells, arginase-dependent metabolism facilitates activation and proliferation through the maintenance of ornithine and ultimately polyamine synthesis that enhances anti-tumour immunity. Similarly, M1 macrophages catabolize arginine through the action of nitric oxide synthase (NOS) to generate nitric oxide (NO), which induces the release of pro-inflammatory cytokines crucial for tumour suppression. Therefore, the arginine polyamine axis may support immune activation in a normal sate. Nevertheless, the TME tends to be immunosuppressive due to overexpression of arginase by tumour-associated macrophages (TAMs) and MDSCs, which direct the flow of arginine towards the production of polyamines at the cost of T-cell function. This metabolism interferes with CD3 complex maturation and antigen recognition leading to defective cytotoxic responses (Refs. 64, 65).

Cytokines like IL-4 and IL-10 foster M2 amplify this loop by promoting M2 polarization and Arg1 upregulation, further driving polyamine synthesis and uptake that sustains immunosuppression (Refs. 66, 67). In this way, cytokine-driven arginine metabolism is adopted by multiple tumour cells, cancer-associated fibroblasts (CAFs), endothelial cells, MDSCs, and M2 macrophages, collectively restricting arginine availability for pro-inflammatory immune cells and enforces tumour tolerance (Figure 3B). Polyamines additionally promote angiogenesis by regulating vascular endothelial growth factor (VEGF) and other pro-angiogenic factors. This supports endothelial proliferation and vascular remodelling central to tumour expansion. Moreover, elevated polyamines support immune evasion by suppressing cytotoxic T and natural killer (NK) cells, while enriching immunosuppressive populations (MDSCs and M2 macrophages), thereby reinforcing immune exclusion. Through secretion of cytokines such as VEGF, IL-6, and TGF-β, polyamines remodel the tumour niche to favour tumour progression and stromal cell activation (Ref. 68).

Contradictory findings show that while polyamines generally contribute to immunosuppression, selective polyamine deprivation can transiently disrupt tumour growth but may also suppress beneficial immune cell functions if not carefully targeted (Refs. 69, 70). Additionally, the dual nature of arginine metabolism means that intervention strategies require precise modulation to balance immune activation and tumour suppression (Refs. 71, 72). Targeting polyamine metabolism in the TME offers therapeutic potential to restore anti-tumour immunity and improve responses to immunotherapy (Ref. 3). Several polyamine synthesis inhibitors under clinical investigation show promise in restoring metabolic balance, improving immunotherapy responsiveness and enhancing immune-mediated tumour clearance (Ref. 73).

#### Metastasis

Beyond immune modulation, polyamines significantly contribute to the metastatic cascade, facilitating tumour cell detachment, invasion and colonization at distant sites. Under hypoxic conditions, cancer cells downregulate adhesion molecules, such as E-cadherin and CD44, a process that is amplified by increased uptake of extracellular polyamines. Spermine, in particular, facilitates cellular detachment and migration in colon cancer cells such as HT-29, potentiating metastatic spread (Ref. 18). Elevated polyamine biosynthesis, often driven by ODC overexpression, promotes matrix metalloproteinase (MMP-2 and MMP-9) expression, enzymes that degrade the extracellular matrix and facilitate local invasion (Ref. 74). Concurrently, polyamines stabilize critical signalling pathways including PI3K/Akt and β-catenin, further driving EMT and enhancing cell motility (Refs. 22, 75).

Pre-clinical models confirm that pharmacologic inhibition of ODC with DFMO downregulates MMP levels and suppresses metastatic dissemination (Refs. 76–78). Additionally, high polyamine levels suppress anti-tumour immunity by inhibiting cytotoxic T-cells and promoting immunosuppressive populations like regulatory T-cells and M2 macrophages (Ref. 3). In Oesophageal Squamous Cell Carcinoma (ESCC), overexpression of ODC correlates with advanced disease and metastasis. **ODC inhibition**, either through gene silencing or DFMO, suppresses proliferation and induces apoptosis by blocking polyamine synthesis. Clinically, targeting polyamine metabolism with DFMO, alone or in combination with polyamine transport inhibitors and immunotherapies, exhibits promising synergistic anti-metastatic effects, positioning it as a promising therapy strategy for ESCC (Ref. 79). Moreover, the study demonstrates that DFMO significantly suppresses Ewing sarcoma cell proliferation and cancer stem cell-like traits *in vitro*, while also preventing tumour growth and metastasis in mouse models. Mechanistically, DFMO induces ferroptosis by depleting intracellular polyamines, rendering metastatic Ewing sarcoma cells highly vulnerable to this oxidative cell death pathway. Transcriptomic and metabolomic analyses confirmed activation of ferroptosis-related genes and lipid peroxidation. These findings highlight polyamine depletion-induced ferroptosis as a potent anti-metastatic mechanism and support clinical evaluation of DFMO as an adjuvant therapy to prevent metastatic recurrence (Ref. 80). Although DFMO-induced ferroptosis suppresses metastasis in Ewing sarcoma, some paradoxical pieces of evidence indicate that, under certain conditions, ferroptosis can also promote tumour progression. Sublethal ferroptotic stress can provoke inflammatory signalling, immune evasion and adaptive metabolic rewiring, thereby making the surviving cancer cells more invasive and resistant to therapies. Though, ferroptosis induced by DFMO exerts anti-metastatic effects, paradoxically, context-dependent ferroptotic responses in other cancers could enhance tumour aggressiveness and escape mechanisms (Refs. 3, 81, 82). Moreover, some tumour types show variable sensitivity to polyamine-targeting therapies, emphasizing the need for tailored approaches (Ref. 81).

### Polyamines in cell death

#### Ferroptosis

Among the different regulated types of cell death, ferroptosis is a newly recognized key mechanism at the crossroads of iron metabolism, lipid peroxidation and redox homeostasis. Ferroptosis process, characterized by an iron-dependent accumulation of reactive oxygen species (ROS) and oxidative stress-driven form of cell death involved in cancer progression, metastasis and resistance to therapy.

Polyamines serve as critical modulators of ferroptosis, shaping how cancer cells balance oxidative stress and survival. They regulate redox homeostasis through the glutathione system and antioxidant defences, impacting lipid peroxidation and iron metabolism, the core elements of ferroptosis (Refs. 83, 84). Interestingly, polyamines, particularly spermidine and spermine, exhibit dual roles in ferroptosis. On the one hand, they enable cancer cells by resisting oxidative stress-induced injury, while on the other hand, they promote ferroptosis by enhancing ROS and lipid peroxidation. Experimental evidence demonstrates that spermine induces lipid peroxidation and ferroptosis in colorectal cancer cells, directly linking polyamine accumulation to oxidative cell death (Refs. 85–87). At the mechanistic level, polyamine catabolism via PAOX and SMOX, and critically, SAT1 generates H_2_O_2_, a potent driver of lipid ROS formation. When intracellular iron levels are elevated, these reactions accelerate Fenton chemistry, intensifying oxidative stress and actively promoting ferroptosis. This process, influenced by iron overload, activates signalling pathways such as WNT/MYC, which upregulate ODC1, creating a feedback loop that exacerbates ferroptosis, Figure 3A (Ref. 83). From a therapeutic perspective, ODC1 inhibitors, such as DFMO, sensitize lung cancer cells to ferroptosis and are emerging as ‘ferroptosis sensitizers (Ref. 64). Conversely, antioxidants and enhanced polyamine-mediated reduction of oxidative stress counteract lipid peroxidation and function as ferroptosis inhibitors, illustrating the dynamic, bidirectional control governed by polyamines.

Clinically, tumours with high ODC1 expression and elevated polyamine levels such as lung adenocarcinoma and pancreatic ductal adenocarcinoma, exhibit aggressive phenotypes, yet simultaneously display increased vulnerability to ferroptosis (Ref. 85). The MUC1-HIF-1α axis in pancreatic cancer modulates polyamine catabolism via SAT1 induction, by re-routing the metabolic flux into the tricarboxylic acid (TCA) cycle, thereby connecting ferroptosis regulation to tumour progression and therapy resistance (Ref. 88). While polyamine depletion promotes ferroptosis and inhibits metastasis, contradictory findings suggest that in certain contexts, polyamines may serve a protective antioxidant role. This underscores the complex, context-dependent roles of the polyamine-ferroptosis axis (Ref. 85). In our opinion, this duality reflects the fine balance between the cytotoxic and cytoprotective functions of polyamines in cancer biology. As demonstrated by Wei *et al.*, activation of the Sp1-SAT1 polyamine catabolic pathway enhances ferroptotic sensitivity in pancreatic ductal adenocarcinoma by promoting ROS accumulation and lipid peroxidation. However, under specific metabolic or hypoxic conditions, elevated polyamine levels may buffer oxidative stress and preserve cell survival, suggesting that therapeutic modulation of polyamine metabolism should be precisely timed and contextually adapted to maximize ferroptotic and anti-metastatic outcomes.

#### Hypoxia

Hypoxia is a pervasive feature of the tumour microenvironment, especially in highly proliferating solid tumours, which provokes widespread cellular adaptations that are primarily orchestrated by hypoxia-inducible factor 1α (HIF-1α) to counter the oxygen demand exceeding the supply. Under normal physiology, polyamine biosynthesis under low oxygen conditions generally drops to save energy, and there is not much involvement in hypoxia adaptation (Ref. 89). However, in cancer cells, the pattern reverses dramatically. Hypoxia stimulates a pronounced increase in polyamine biosynthesis and uptake, chiefly via induction of ODC and transport systems, leading to elevated intracellular polyamines that robustly support tumour survival and progression. Mechanistically, polyamine accumulation stabilizes HIF-1α during hypoxia, protecting it from ubiquitin-mediated degradation, thereby enabling sustained transcriptional activation of genes promoting angiogenesis, such as vascular endothelial growth factor (VEGF), which facilitates neovascularization critical for tumour expansion, Figure 3B (Ref. 90). Concurrently, polyamines induce epithelial-mesenchymal transition (EMT) by upregulating transcription factors, including Snail, Twist and ZEB1, which suppress epithelial markers such as E-cadherin and promote mesenchymal traits, thereby enhancing the migratory and invasive capabilities of cancer cells. This significantly contributes to metastasis, particularly in the hypoxic niches of breast, liver and pancreatic cancers (Ref. 91).

Beyond promoting invasion, polyamines render cells resistance to apoptosis. On a mechanistic level, polyamines stabilize anti-apoptotic proteins and inhibit caspase-3 and Bax, activation, two key effectors of programmed cell death, supporting chemoresistance (Ref. 92). On metabolic level, they enhance aerobic glycolysis, also known as the Warburg effect, bestowing a metabolic advantage under oxygen-deprived conditions. They also have a profound impact within the hypoxic tumour microenvironment by modulating immune-suppressive cells, such as MDSCs and TAMs, which promote immune evasion and therapeutic resistance (Ref. 93). Interestingly, not all polyamine functions in hypoxia are uniformly protective. In certain contexts, polyamine depletion sensitizes tumour cells to hypoxia-induced oxidative stress and cell death, indicating that polyamines have a dual, context-dependent role under hypoxia (Ref. 90). Notably, when polyamines were depleted during hypoxia, cancer cells showed increased apoptosis, highlighting that polyamines play a protective role in hypoxic adaptation but also reveal a therapeutic potential vulnerability. Study demonstrated that hypoxia induces robust upregulation of ornithine decarboxylase (ODC) and polyamine transport genes through **HIF-1α–dependent signalling**, thereby enhancing intracellular polyamine accumulation and promoting tumour survival under oxygen deprivation. In our opinion, this dual role suggests that targeting polyamine metabolism in the hypoxic tumour microenvironment could be a highly promising strategy; by inhibiting polyamine synthesis or uptake, one may exploit the hypoxia-induced dependency of tumour cells and tip the balance towards cell death. Thus, combining ODC inhibitors like DFMO with agents that exacerbate hypoxic stress (e.g. anti-angiogenic therapy) could provide a clinically meaningful strategy.

#### Efferocytosis

Efferocytosis is the process by which macrophages clear apoptotic cells, profoundly influencing tumour immunity and disease progression. Within the tumour microenvironment, macrophages frequently polarize towards two distinct phenotypes (M1 and M2). M1 macrophages exert pro-inflammatory and tumour-suppressing functions, and M2 macrophages exert anti-inflammatory and tumour-promoting phenotypes. Mostly, M2 macrophages exhibit enhanced efferocytosis capacity, promoting tumour progression through immunosuppressive functions (Ref. 94).

Recent research evidence highlights a critical link between efferocytosis and polyamine metabolism in TAMs. Instead of relying on *de novo* synthesis, TAMs accumulate arginine-derived polyamines, particularly spermidine and spermine, via Rac1-dependent uptake after ingesting apoptotic cells. This accumulation suppresses inflammatory cytokines such as IL-1β and IL-6, reinforcing an immunosuppressive phenotype (Refs. 95, 96). In M2 macrophages, Arginase 1 (Arg1) activity converts arginine to putrescine, subsequently activating Rac1 guanine nucleotide exchange factor (GEF) Dbl, sustaining Rac1 signalling in a feed-forward loop-driven efferocytosis (Refs. 95, 97). Polyamines further polarize macrophages towards an M2-like phenotype. Spermidine activates AMP-activated protein kinase (AMPK) to boost oxidative phosphorylation and IL-10 production, while spermine suppresses pro-inflammatory iNOS and TNF-α expression establishing an immunoregulatory state (Refs. 98, 99). Moreover, high polyamine levels in IL-4 treated macrophages induce M2 marker genes independent of Arg1, concurrently suppressing pro-inflammatory genes in LPS-stimulated macrophages (Ref. 100). Cancer takes advantage of this metabolic axis. As an example, IL-33 induced ODC expression in oesophageal cancer promotes polyamine biosynthesis in TAMs and sustains their M2-like immunosuppressive phenotype marked by PDL1 and CTLA4 and recruits regulatory T-cells, which suppress Th1 responses (Refs. 58, 99). Similarly, glioblastoma TAMs increase polyamine production to buffer the acidic microenvironment, thereby facilitating tumour cell survival. Inhibition of polyamine synthesis or uptake (e.g. with DFMO) interrupts tumour growth by restoring T-cell immunity (Refs. 96, 98).

**Figure 4.**
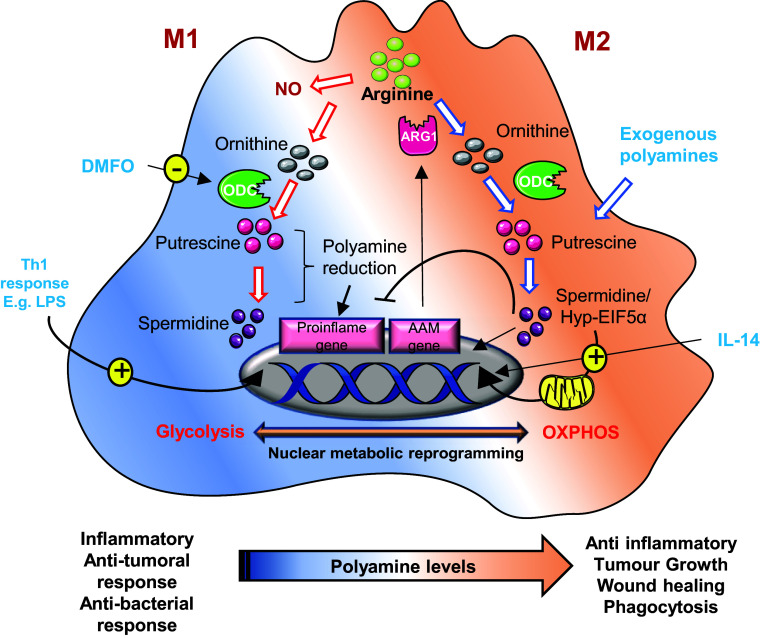
Schematic representation of polyamine metabolism on M1 and M2 macrophage polarization: In M2 (alternatively activated) macrophages, by suppressing pro-inflammatory genes and AAM genes, activate anti-inflammatory genes like arginase-1 (ARG1), promoting tumour immune evasion. Polyamines are crucial for this process; their synthesis is upregulated by IL-14. These macrophages rely on oxidative phosphorylation, a process regulated by hypusinated eIF5A (hyp-eIF5A), which enhances mitochondrial protein expression. In contrast, M1 macrophages, activated by Th1 cytokines, produce nitric oxide (NO) and inflammatory cytokines, utilizing arginine for NO synthesis. Uptake of external polyamines supports M2 polarization, while genetic or chemical inhibition of ornithine decarboxylase (ODC) a key enzyme in polyamine production shifts macrophages towards the pro-inflammatory M1 state. Thus, mitochondrial metabolism and polyamine availability are key regulators of macrophage function and phenotype.

Polyamines also interface directly with efferocytosis signalling pathways. Spermidine acts as a chemoattractant or ‘find-me’ signal guiding macrophages towards dying cells via purinergic receptor activation, while phagocytic receptor activation (MerTK/AXL) induces Rac1-driven actin remodelling critical for apoptotic engulfment (Refs. 97, 101). Efficient phago-lysosomal fusion and acidification, essential for tumour-promoting efferocytosis, are regulated by the CFTR–Arg1 axis, as demonstrated in metastatic pancreatic cancer (Ref. 102). At the metabolic level, a shift in M2 macrophages towards fatty acid oxidation and mitochondrial oxidative phosphorylation (OXPHOS) is supported by spermidine-mediated increases in IL-4, which hypusinate eIF5A, a vital process for mitochondrial function and translation. Inhibiting hypusination impairs both OXPHOS and efferocytosis, reducing tumour-supportive macrophage activity (Figure 4) (Refs. 103, 104).

Interestingly, while polyamine accumulation during efferocytosis often supports tissue repair, it may also undermine anti-tumour immunity by promoting the anti-inflammatory, pro-resolving phenotype of macrophages (via putrescine/spermidine produced by the polyamine-ferroptosis axis) and suppressing inflammatory T-cell activation (Ref. 61). Yet, macrophage polarization is highly flexible depending on tumour type, microenvironmental cues (hypoxia, metabolic stress) and systemic factors (age, diet and circulating polyamines); the functional outcome of efferocytosis-driven polyamine accumulation can flip; in some settings it fosters tumour tolerance, in others it may limit tumour growth or even promote inflammation. Moreover, elevated systemic polyamine levels could modulate efferocytosis efficacy and macrophage immunoregulatory behaviour at the organismal level, linking metabolism to tumour immunity. In our opinion, this duality underscores the need to contextualize polyamine-targeting therapies in a tumour microenvironment rich in apoptotic debris and efferocytosis activity; polyamine blockade might restore anti-tumour immunity, whereas in other contexts, it might inadvertently impair tissue repair or provoke deleterious inflammation.

### Polyamines in therapy resistance

Resistance to therapy continues to be a significant hindrance to successful cancer treatment, usually leading to relapse of disease, metastasis and poor outcomes. Emerging evidence highlights that polyamine metabolism, through its multifaceted role in epigenetic regulation, redox balance and translational control, is a key determinant of resistance in various types of tumours. Through facilitating cancer stemness, controlling stress responses and remodelling the tumour microenvironment, polyamines play a role in adaptive and acquired mechanisms of drug resistance that undermine therapeutic effectiveness.

#### Cancer stem cells (CSCs)

Cancer stem cells (CSCs) represent a subpopulation endowed with self-renewal, pluripotency and metabolic plasticity, driving tumour heterogeneity, recurrence and therapeutic resistance. Polyamines play a critical role in supporting these CSC phenotypes by modulating epigenetic regulation, translation efficiency and metabolic pathways. For instance, studies by Tamari *et al*. demonstrated that CSCs display elevated polyamine flux, which inhibits Lysine-Specific Demethylase-1 (LSD1), preserving stemness-associated factors like inhibitor of differentiation-1 (ID1) reinforcing stemness (Ref. 105). Moreover, Rondeau *et al*.’s study showed that polyamines required for the eIF5A hypusination play a critical role in leukaemia CSC survival based on spermidine metabolism. eIF5A activity driven by spermidine, and its downstream targets, such as KAT7, connects translational regulation to stem cell maintenance (Ref. 106).

Targeting polyamine metabolism by either ODC inhibition (DFMO) or interference with the hypusination pathway provokes CSC differentiation and diminishes clonogenic potential. Mechanistically, polyamine metabolism interacts with various key stemness-regulating networks, notably the Lin28/let-7 and Wnt/β-catenin pathways, thereby setting up self-reinforcing feed-forward loops that maintain CSC phenotypes. Polyamine-dependent hypusination of eIF5A hyperactivates Lin28B expression, represses let-7 microRNAs and increases glycolytic flux, collectively maintaining a stem-like metabolic state, Figure 3D (Refs. 107, 108).

However, this relationship is highly context-dependent. While, increased polyamine flux typically supports CSC survival, self-renewal and therapeutic resistance, aberrant or excessive polyamine activities may become detrimental and provoke either metabolic stress or differentiation. For example, Lozier *et al.* (Ref. 109) observed that DFMO-dependent ODC inhibition represses the Lin28/let-7 axis and inhibits glycolytic CSC traits in neuroblastoma (Ref. 109). These findings highlight the metabolic adaptability of CSCs, suggesting that monotherapies targeting polyamine synthesis may provide transient benefits. Consequently, combinatorial strategies such as DFMO with polyamine transport inhibitor AMXT1501 or agents targeting redox and immune metabolism represent more promising approaches to eradicate CSC populations and prevent tumour relapse.

#### Drug resistance

Across a range of tumour types, polyamine metabolism has a complex and bidirectional interaction with cancer therapy resistance. Drug-resistant phenotypes in bladder cancer cells (T24/THP) show decreased levels of polyamines and important biosynthesis enzymes such as SRM and ODC1 (Ref. 110). These enzymes play a part in drug-sensitive cells; their overexpression restores resistance while their silencing enhances chemosensitivity. Additionally, by causing metabolic reprogramming, excessive depletion of polyamines has the potential to induce therapeutic resistance, indicating a highly specific and balance-sensitive connection between polyamine flux and response to medication. Silencing these enzymes in drug-sensitive cells enhances chemosensitivity, whereas overexpression re-establishes resistance, implicating a drug tolerance through c-MYC-dependent regulation. In addition, excessive polyamine depletion may also contribute to therapy resistance through triggering metabolic reprogramming, suggesting a balance-sensitive and highly specific relationship between polyamine flux and drug response.

In melanoma, pancreatic cancer and TNBC, increased activities of AMD1 and SAT1 strengthen mitochondrial bioenergetics and redox stability and hence supporting drug resistance. Inhibition of polyamine synthesis with DFMO or utilization of polyamine analogues can re-sensitize these tumours to drug treatment by mitochondrial dysfunction (Ref. 8). Similarly, in colorectal cancer, polyamines foster an immunosuppressive microenvironment. The study depicts that adenosine deaminase acting on RNA (ADAR1-mediated RNA) editing of AZIN1 increases ODC activity and polyamine levels, which induce M2 macrophages that drive tumour evasion and resistance to oxaliplatin chemotherapy (Ref. 111).

In breast cancer, contrasting drug resistance mechanisms were found. Although curcumin impacts polyamine metabolism by inducing G2/M arrest via PI3K and NFκB inhibition, these effects relate more to cell cycle control than direct drug resistance. Chemoresistance in breast cancer is more strongly linked to proteins like P-glycoprotein and splicing factor 3A subunit 2 (SF3A2) than polyamine dynamics (Ref. 112). Additionally, while polyamines are associated with Poly (ADP-ribose) Polymerase (PARP) activity, there is a negligible mechanistic link to PARP inhibitor resistance, which is mainly driven by BRCA mutations, alternative DNA repair pathways and efflux transporter expression (Ref. 113). Current strategies to overcome PARP resistance focus on dosing optimization and combination therapies rather than targeting polyamine metabolism.

From a metabolic perspective, polyamines also modulate redox balance and ROS, suggesting a connection between oxidative defence and drug resistance. Combining approaches that integrate polyamine depletion with agents targeting redox pathways may offer synergistic benefits (Ref. 114). Overall, polyamine metabolism underlies multiple mechanisms of therapy resistance, ranging from mitochondrial resilience to microenvironmental modulation. Table 1 consolidates the polyamine-driven resistance pathways in various cancer types. It is insightful to appreciate multifaceted roles of polyamine metabolism in drug resistance and unlock novel strategies to overcoming therapy failure. Inhibition of polyamine biosynthesis, catabolism or transport can be used complimentarily with current treatments, potentially reversing resistance and enhancing patient outcomes. Nonetheless, given the contradictory evidence, personalized therapeutic strategies considering tumour context and polyamine dynamics are essential.

**Table 1.** Polyamine-mediated mechanisms of resistance to cancer therapies across different cancer types

Cancer type	Cell lines	Involvement of the polyamine pathway	Molecular mechanism	Outcome	Therapeutic strategy	References
Melanoma	HEK293T, SK-mel–28, & Hs294T cells	Putrescine and spermidine	Increased mitochondrial protein translation & OXPHOS. EIF5A hypusination	BRAF inhibitor resistance (Vemurafenib)	Targeting ODC1/AMD1 (e.g., DMFO) and block hypusination (e.g., with GC–7)	(Refs. 115, 116)
Lung Cancer	A549 (TCHu150), H1299 (TCHu160), PC9 (SCSP–5085), H23 (SCSP–581), fibrosarcoma cell line HT1080 (TCHu170)	Polyamines (Spermine) activates the WNT/MYC/ODC1 pathway	Ferroptosis induction	Increased efficacy of radiotherapy and cisplatin	Use Radiation-therapy or cisplatin; block ferroptosis with inhibitors (Fer–1)	(Ref. 83)
Hepatocellular Carcinoma (HCC)	Human HCC cell lines, HepG2, Huh7, and MHCC97H cells	↑ PAscore	Increased immune dysfunction (↑ TIDE). ↑ CSC index	Decreased sensitivity to immunotherapy and chemotherapies (e.g., 5-FU, lapatinib)	Combining polyamine targeting agents with checkpoint inhibitors or use of sorafenib/oxaliplatin/gemcitabine in high-PAscore patients	(Refs. 117, 118)
Osteosarcoma	HOS, 143B, HOS-MNNG, U2OS, MG63, MG63.2 and SJSA	Polyamines promote activation of the YAP1-regulated glutamine metabolic pathway	YAP1 phosphorylation and nuclear translocation. Increased glutaminase (GLS) and glutathione	DFMO resistance	Co-target YAP1 or glutaminase with DFMO to overcome adaptive resistance	(Ref. 119)
Breast Cancer (MCF–7/ADR)	MCF–7/ADR and HUVEC cells	High ODC activity elevates polyamines, promoting cancer progression via ODC, MMPs, MAPK/Erk, and VEGF	Ursolic acid binds to ODC active site	Decreased adhesion and migration in drug-resistant cells	Use of UA or derivatives to inhibit ODC	(Ref. 120)
Breast Cancer (TNBC)	MDA-MB–231& Hs 578 T	Methionine enhances polyamine biosynthesis	Increased SAM	Therapy resistance	Use of methionine restriction or inhibit SAM-dependent enzymes	(Ref. 121)
Multiple cancers	Human neuroblastoma cell lines SMS-KCNR, SH-SY5Y, BE-(2)C	High ODC activity elevates polyamines, promoting cancer progression via ODC, MMPs, MAPK/Erk, and VEGF	Inhibition of ODC Polyamine transport (from diet/microbiome	Both chemo resistance and radiotherapy resistance	Combine DFMO with polyamine transport inhibitors (e.g., AMXT–1501)	(Ref. 122)
DNA repair-driven resistance	Human neuroblastoma cell lines SMS-KCNR, SH-SY5Y, BE-(2)C	Elevated polyamines boost PARP1 activity, enhancing DNA repair and conferring resistance to DNA damaging chemotherapies	Enhanced PARP1 activity	Resistance to genotoxic chemotherapy (e.g., platinum agents)	Combine polyamine depletion with PARP inhibitors; use polyamine analogues/nano polyamines	(Ref. 122)

#### Polyploid Giant Cancer Cells (PGCCs)

There exists a rare population of cancer cells known as PGCCs that are distinguished by their expanded size and aberrant nuclear content. These cells are frequently the consequence of stress-induced events like endoreplication, cell fusion or failed cytokinesis. These atypical cells have characteristics of stem cells, which aid in tumour recurrence, metastasis and resistance to therapy. Aneuploid offspring of greater genetic heterogeneity and invasive capacity are generated when diploid tumour cells experience an altered or giant cell cycle, consisting of phases of genome duplication without traditional mitosis. PGCCs are often caused by genotoxic stressors such as radiation, chemotherapy, hypoxia or environmental exposures. They are associated with changes in the tumour microenvironment and mutations in genes including SF3B1 and TP53 (Ref. 123).

Polyamines intersect with PGCC biology at multiple regulatory levels. Spermidine and spermine induce the formation of multinucleated giant cells in normal tissues and stimulate mTOR signalling, a key driver of cell growth and multinucleation, paralleling PGCC formation under stress (Refs. 124, 125). Polyamines modulate autophagy and oxidative stress responses, which are essential for PGCC survival and proliferative capacity (Ref. 124, 126). Mechanistically, elevated polyamines stabilize the expanded genome by condensing DNA and modifying chromatin architecture while impacting mitochondrial function. Interestingly, spermine can also trigger the release of cytochrome c and potentially prime mitochondrial-mediated cell death pathways (Refs. 99, 126, 127). These effects suggest a dual role supporting PGCC survival and stemness at moderate levels but promoting lethality under metabolic overload.

Clinically, PGCC presence strongly correlates with high histologic grade, poor prognosis and therapeutic refractoriness, most notably observed in prostate, breast, lung and colon cancers (Refs. 123, 128). According to transcriptomic research, distinct gene profiles associated with invasiveness and recurrence are expressed by tumour populations driven by PGCC, and these aggressive subpopulations are exacerbated by dysregulated polyamine metabolism. The delicate balance regulating PGCC behaviour is highlighted by contradictory studies showing that although polyamines support PGCC development and survival, excessive polyamine accumulation may cause cytotoxic stress (Refs. 123, 129). Novel insights into tumour heterogeneity, resistance mechanisms and possible treatment targets aimed at eliminating these robust cancer cell populations can be gained by comprehending the interaction between polyamine metabolism and PGCC biology.

## Therapeutic potential of polyamines

Given the critical roles of polyamines in cancer cell proliferation, stress adaptation and immune modulation, polyamine metabolism has emerged as a promising therapeutic target. Several strategies have evolved to block biosynthesis flux, block transporters and induce catalytic cytotoxicity. Among these methods, irreversible inhibition of ODC, by drugs like DFMO, is still the most advanced approach (Ref. 130). Table 2 comprehensively summarizes polyamine-targeting drugs and combination strategies for their therapeutic potential in clinical trials.

**Table 2.** A summary of polyamine-targeting agents, their mechanisms and clinical trial status

Drug	Dose/ROA	Mechanism of action	Duration status	Intervention	Study status	Primary objective	Outcome measure	Study ID	References
DFMO (Eflornithine)	Oral DFMO at a dose of 500 to 1000 mg/m^2^ BID (on each day of 28-day cycle)	Irreversible inhibitor of ODC, blocking polyamine synthesis	27 cycles, each cycle 28 days (completed study)	N/A	Phase-II	Long-term survival in high-risk neuroblastoma	Number of Participants With Event-Free Survival (EFS) During Study.	NCT02395666	(Refs. 131, 132)
AMXT1501	AMXT1501:1200 mg BID-oral 28-day cycle. DFMO: 2 ml/hr,IV continuous infusion	Competitive inhibitor of **polyamine uptake transporter system**; blocks extracellular polyamine import.	28-day cycle	AMXT1501, DFMO	Phase I/II Terminated.	Determines Dose Limiting Toxicities and RP2Ds in AMXT 1501 in combination with IV DFMO	Required re-formulation of DFMO from IV to a capsule to maintain safety	NCT05500508	(Ref. 133)
AMXT1501+ DFMO	Experiment part:1 Starting: 80 mg orally, once daily (days 1–14 fasted), then combined with DFMO (day 15–28). DFMO dosing: 250 mg BID orally (day 15–28). Experiment part:2 starting both drugs 500 mg BID orally in the morning, DFMO alone before bedtime 28 day cycle	Dual inhibition of **biosynthesis + transport**; depletes intracellular polyamines and prevents compensation.	28 day cycle, repeated cycles at investigator discretion (2-years study)	AMXT1501, DFMO	Phase-I(completed)	Establish safety and dose level of AMXT 1501 dicaprate alone, and in combination with DFMO, in cancer patients.	Determine Dose Limiting Toxicities and Recommended Phase2 Dose in AMXT 1501 in combination with DFMO	NCT03536728	(Ref. 7)
SAM486A	IV with 100 mg/m^2^ SAM486A as a daily 1-h infusion for 5 days repeated every 3 weeks.	Inhibitor of **S-adenosylmethionine decarboxylase**	Total–8 cycles	NA	Terminated/inactive	To investigate toxicity	Demonstrated concept validity but lacked tumor selectivity, Reason: Anemia was the primary hematological toxicity, thrombocytopenia	NA	(Ref. 134)
SBP101 (spermidine analogue)	SBP101-SC nab-paclitaxel-IV Gemcitabine Injection-IV	Mimics spermidine to disrupt polyamine-dependent translation and redox homeostasis	Total 24 months	SBP–101, nab paclitaxel, Gemcitabine Injection	Phase-I(completed)	To assess the safety, tolerability and pharmacokinetics of SBP–101 when combined with nab-paclitaxel and gemcitabine in subjects with previously untreated metastatic pancreatic ductal adenocarcinoma and to identify a recommended phase 2 dose	Combined with gemcitabine/nab-paclitaxel; promising early efficacy; ocular toxicity under monitoring	NCT03412799	(Refs. 133, 135)
PG–11047	50–750 mg the MTD was 610 mg weekly (IV infusion)	**PG–11047** is a polyamine analogue that disrupts polyamine metabolism	1,8 and 15 of a 28-day cycles (2 cycles total 8 weeks)	N/A	Phase-I	Study investigates PG–11047 in patients with advanced refractory metastatic solid tumors	Maximum Tolerated Dose	NCT00705653	(Ref. 136)
SL–11047	IV over 30 minutes		Days 1–5 of each cycle of 21 days cycles repeat every 21 days	N/A	Phase–1	To determine MTD and characterize the toxicity profile and evaluate preliminary anti tumor activity	MTD	NCT00293488	(Ref. 137)
DFMO+Sulindac	Difluoromethylornithine – 500 mg oral for 14 days, combined with sulindac for 15 days Sulindac – 150 mg oral for 15 days, combined with DFMO for 14 days	**DFMO** irreversibly inhibits **ODC**, while **Sulindac**, is inhibits **COX enzymes** there by reduces prostaglandin-mediated induction of ODC and tumour-promoting inflammation.	NA	Difluorom ethylornithine Sulindac	Phase-II (withdrawn)	Measure intra-subject urine N1-monoacetylspermidine and dcSAM variability during the pre-drug phase	Reason for withdrawn: Funding and staffing	NCT01636128	(Ref. 138)
DFMO+ Testosterone+ Enzalutamide	DFMO: 1000 mg BID, oral in Days 1–7: Alone, Days 8–63: With testosterone (IM) Testosterone-cypionate: 400 mg (high-dose), Day 8 and Day 36(2 doses per 119-day cycle) Enzalutamide:160 mg Oral (PO) once a day (QD), Days 64–119	DFMO inhibiting ODC, and **testosterone** activates androgen receptor while **enzalutamide** blocks AR activation to counter testosterone effect. In Combined, **DFMO + enzalutamide** synergistically suppress **androgen-driven polyamine production and tumour growth**, while **testosterone** provides a controlled AR stimulus to assess pathway dependence	119 day cycle	DFMO, testosterone cypionate, Luteinizing hormone-releasing hormone (LHRH) analogue, Enzalutamide	Phase-II (recruiting)	PSA response Proportion of participants achieving ≥50% decline in prostate-specific antigen (PSA) from baseline, assessed at Cycle 1 Day 64 (119-day treatment cycles).	PSA response rate at Cycle 1 Day 64	NCT06059118	(Ref. 139)
Celecoxib+Cyclophosphamide	Oral DFMO + celecoxib (14 days/cycle) with a combination of IV cyclophosphamide + topotecan; 7-day DFMO/celecoxib lead-in for polyamine depletion up to 17 cycles if tolerated and no progression.	DFMO inhibiting ODC, Celecoxib suppresses COX–2–driven polyamine production and inflammation. Cyclophosphamide causes DNA damage	17 cycles, each cycle has 26 days	Celecoxib, cyclophosphamide	Phase-I (completed)	To find the highest dose of DFMO that can be given with celecoxib, cyclophosphamide and topotecan without causing severe side effects.	Number of participants with adverse events as a measure of safety and tolerability.	NCT02030964	(Ref. 140)
Eflornithine+ Bicalutamide	Oral eflornithine and oral bicalutamide once daily	**Eflornithine** inhibits ODC to deplete polyamines, while **Bicalutamide** inhibits AR-driven ODC1 expression. Together, they **suppress androgen-stimulated polyamine synthesis**, reducing tumour growth	11 months	Eflornithine Bicalutamide	Phase-II (completed)	To compare intra-tumoral levels of polyamines in patients with localized prostate cancer	Mean polyamine levels decreased most with eflornithine **+ bicalutamide alone** (spermine ↓70%, spermidine ↓60%, putrescine ↓50%), moderately with **eflornithine alone** (↓30–40%), minimally with **bicalutamide alone** (<10%), and were unchanged in controls, confirming **additive polyamine inhibition**	NCT00086736	(Ref. 141)

### Targeting polyamine biosynthesis

Targeting polyamine metabolism remains one of the most established metabolic approaches in oncology, historically led by DFMO. DFMO has shown the clearest success in chemoprevention, particularly in low-dose DFMO combined with sulindac, which has reduced recurrent colorectal adenomas in randomized trials and meta-analyses, supporting a chemopreventive role in high-risk patients (Ref. 142). In conventional oncology, DFMO has shown limited efficacy in single-agent trials, producing only limited objective responses in advanced solid tumours. However, modern trials have refocused DFMO as a cytostatic, maintenance or combinatorial agent, notably in neuroblastoma, where recent regulatory discussions and maintenance studies have shown benefit (Ref. 143). A major limitation of DFMO monotherapy is the compensatory increase in polyamine uptake. Cancer cells upregulate polyamine transporters after blockade of biosynthesis, thereby undermining the efficacy of DFMO. To address this limitation, AMXT1501, a clinical polyamine transport inhibitor, has been developed. Co-administration with AMXT1501 with DFMO effectively prevents compensatory replenishment and has advanced into Phase I studies, receiving orphan-drug designation in paediatric indications such as neuroblastoma. Early clinical reports indicate acceptable tolerability and pharmacodynamic polyamine depletion when AMXT1501 is paired with DFMO (Ref. 7).

Additional strategies include the modulation of polyamine catabolism, which offers complementary therapeutic avenues. Enzymes such as SMOX and SSAT control intracellular polyamines biosynthesis and redox balance. Therefore, targeting these enzymes can alter the oxidative balance or induce cell cytotoxicity through generation of H_2_O_2_. Given the enhanced polyamine uptake capacity observed in cancer cells, targeting polyamine transport systems represents another promising therapeutic strategy (Ref. 35). As discussed earlier, dual inhibition with combination therapies of DFMO and AMXT1501 has also shown synergistic efficacy, hindering intracellular replenishment and mitigating the sensitivity of cancer cells to chemotherapy and immunotherapy (Ref. 122). Moreover, the MYC-ODC1 axis, which transcriptionally coordinates the expression of polyamine biosynthetic enzymes, has emerged as a high-priority target. The MYC oncogene directly regulates polyamine metabolism by controlling enzymes like ODC1. Inhibiting the MYC-ODC1 axis is proposed as a therapeutic strategy, especially in MYC-overexpressing cancers (Ref. 144).

Synthetic polyamine analogues, such as N^1^, N^11^-diethylnorspermine (DENSpm), act as structural mimetics that deregulate homeostatic feedback. They catabolically induce SSAT and SMOX, leading to excessive polyamine depletion by oxidation and increased ROS production, selectively inducing cytotoxicity in tumour cells (Ref. 46). Likewise, using RNA interference (RNAi) technology had showed promising results. Silencing of the key polyamine biosynthetic enzymes, spermidine synthase (SRM), S-adenosylmethionine decarboxylase and ODC MCF-7 breast cancer cells, induces growth inhibition, cell death, cell cycle arrest and downregulation of proliferation-associated genes (Ref. 145). Combination therapies integrating polyamine pathway inhibitors with drugs targeting mTOR, MYC or immune checkpoints demonstrate promising synergistic effects and synthetic lethality in resistant cancers (Ref. 146). Co-treatment of DFMO with rapamycin (mTOR inhibitor) induces additive cytotoxicity and enhanced translation inhibition in breast cancer cells, suggesting dual inhibition of polyamine and mTOR pathways and providing therapeutic benefits. In MYC-driven neuroblastoma, DFMO combined with AMD1 inhibitor (SAM486) potently block tumour initiation in MYCN transgenic mice (Refs. 144, 147), understanding synthetic lethal interactions between MYC and polyamine metabolism, while triple combination with celecoxib regresses established tumours harbouring MYCN amplification and TP53 mutations (Ref. 147). Furthermore, DFMO combined with α-PD1 checkpoint blockade achieves complete tumour regression in 40% of treated mice through CD8^+^ T-cell dependent mechanism, significantly enhancing intratumoural CD8^+^ T-cell viability and producing pro-inflammatory cytokine profiles, whereas monotherapies yielded minimal responses (Ref. 60). Polyamines-mediate immunosuppression by decreasing MDSCs, Tregs and M2 macrophages while increase activated CD8+ T-cells, providing a mechanistic foundation for checkpoint inhibitor combinations (Ref. 148). In TNBC, blocking *de novo* polyamine synthesis with DFMO re-sensitizes cells to standard chemotherapy and mTOR inhibition (Ref. 125). Thus, polyamine blockade overcomes intrinsic chemoresistance by disrupting translation initiation and targeting synthetic lethal metabolic vulnerabilities (Ref. 149).

### Polyamines as biomarkers

Beyond their metabolic functions, polyamines and related metabolites have emerged as valuable diagnostic and prognostic biomarkers. Elevated levels of polyamines have been detected in multiple biological matrices, including blood, urine and saliva, from patients tissue biopsies across cancers of breast, colorectal, prostate, pancreatic, lung, liver and head-and-neck cancers.

Recent multi-omics analyses have identified polyamine metabolism-related gene signatures that predict prognosis and immunotherapy efficacy in breast cancer and colorectal cancer. Prominent genes, such as ornithine decarboxylase antizyme 1 (OAZ1), SRM, SMOX and SMS, independently correlate with prognostic factors and immune infiltration patterns. At the metabolic level, N-acetylated derivatives (N-AcPUT, N1-AcSPD, N8-AcSPD) have been identified as potential non-invasive biomarkers, with significantly altered concentrations observed in the saliva and urine of patients with head and neck cancer compared to healthy controls. Similarly, urinary polyamine profiles have shown promise as biomarkers for ovarian and prostate cancers, correlating with disease presence and progression. Furthermore, polyamine-related gene indices can stratify cancer patients into high-risk and low-risk groups, with the high-risk group exhibiting poorer clinical outcomes, distinct immune microenvironments and differential drug sensitivities (Refs. 150–154). Collectively, these findings highlight the translational potential of polyamine-centred metabolomics in clinical oncology.

Despite their promise, the use of polyamines as biomarkers and metabolic targets faces significant analytical and methodological challenges. Quantification methods commonly employ liquid chromatography-mass spectrometry (LC–MS/MS) or high-performance liquid chromatography (HPLC), often requiring derivatization (e.g. with dansyl chloride, isobutyl chloroformate or benzoyl chloride) to enhance sensitivity and retention (Ref. 155). However, such steps add analytical complexity, prolonged processing time and potential variability. In contrast, underivatized methods using hydrophilic interaction liquid chromatography (HILIC) or ion-pairing reagents are plagued by low sensitivity, ion suppression and instrument contamination. Sample matrix effects, particularly in complex biological fluids like serum and urine, can lead to inaccurate polyamine estimations, necessitating rigorous validation and internal standardization. Moreover, a lack of comprehensive methods for quantifying both polyamines and their precursors/metabolites (e.g. ornithine, arginine and acetylated derivatives) limits the understanding of polyamine homeostasis (Refs. 155–157).

Current *in vivo* imaging modalities specific to polyamine metabolism remain underdeveloped. While PET probes targeting polyamine transport systems have been developed, their clinical translation remains in early stages. MR spectroscopy can detect polyamines in certain tissues like the prostate, but sensitivity and specificity challenges limit routine diagnostic use (Refs. 158, 159). Addressing these methodological gaps through the development of robust, high-throughput, matrix-independent analytical platforms and validated imaging techniques is essential to fully realize polyamines potential as metabolic biomarkers and therapeutic targets in cancer.

## Conclusion and future perspectives

Polyamines, erstwhile humble molecular guardians of cellular homeostasis, have evolved to become master orchestrators of cancers most formidable traits. The review described in depth of their evolution from their critical role in cell growth and survival to dysregulation, which is intricately entangled with cancer initiation, disease progression and therapy resistance. Their effects span multiple cancer hallmarks from promoting cell growth and angiogenesis to regulation of ferroptosis, immune evasion and PGCC-driven relapse. Targeting polyamine metabolism thus presents a novel therapeutic option. Inhibition of biosynthesis, modification of catabolic flow or inhibition of transport mechanisms has thus proven an attractive therapeutic strategy. Even with promising advancements, significant translational gaps persist. Existing inhibitors are generally not selective for tumour-specific transporters, resulting in systemic toxicity and limited clinical activity. Future research should focus on the development of selective polyamine transport inhibitors and dual-targeting approaches, which concomitantly block biosynthesis and inhibit uptake to abrogate metabolic compensation. Furthermore, the discipline is in need of urgent non-invasive polyamine imaging biomarkers, such as PET tracers or sophisticated mass spectrometry-based probes, to image polyamine fluxes *in vivo* and facilitate patient stratification. Such diagnostic techniques may revolutionize the monitoring of therapeutic responses as well as the identification of vulnerable tumours.

Polyamine-targeted therapy in combination with immunotherapy is another attractive new frontier. As polyamines modulate the tumour immune interface, suppressing cytotoxic T-cell activity and inducing myeloid-derived suppressor cell growth, addition of DFMO or AMXT1501 to immune checkpoint blockade (e.g. compensatory polyamine uptake, anti-PD-1 PD-1/PD-L1) could enhance anti-tumour immunity. Likewise, clarification of how polyamine metabolism is central to the intersection of ferroptosis, hypoxia and the tumour microenvironment will yield novel combination treatments. In conclusion, the biology of polyamines presents both vulnerabilities and opportunities, as its complexity constrains therapeutic design to be context-dependent. Advancing this field will require a systems-level understanding of polyamine flux, improved selectivity of metabolic inhibitors, biomarker-driven clinical trial design and strategic integration with immune and redox-based therapies. Closing these pre-clinical, clinical gaps might finally convert the long-recognized significance of polyamines into concrete precision oncology outputs.

## Data Availability

No data was used for the research described in the article.
